# Influence of custom-made and prefabricated insoles before and after an intense run

**DOI:** 10.1371/journal.pone.0173179

**Published:** 2017-02-28

**Authors:** Angel Gabriel Lucas-Cuevas, Andrés Camacho-García, Raúl Llinares, Jose Ignacio Priego Quesada, Salvador Llana-Belloch, Pedro Pérez-Soriano

**Affiliations:** 1 Research Group in Sport Biomechanics (GIBD), Department of Physical Education and Sports, University of Valencia, Valencia, Spain; 2 Polytechnic University of Valencia, Alcoy, Spain; 3 Biophysics and medical physics group, Department of Physiology, University of Valencia, Valencia, Spain; Nanyang Technological University, SINGAPORE

## Abstract

Each time the foot contacts the ground during running there is a rapid deceleration that results in a shock wave that is transmitted from the foot to the head. The fatigue of the musculoskeletal system during running may decrease the ability of the body to absorb those shock waves and increase the risk of injury. Insoles are commonly prescribed to prevent injuries, and both custom-made and prefabricated insoles have been observed to reduce shock accelerations during running. However, no study to date has included a direct comparison of their behaviour measured over the same group of athletes, and therefore great controversy still exists regarding their effectiveness in reducing impact loading during running. The aim of the study was to analyse the acute differences in stride and shock parameters while running on a treadmill with custom-made and prefabricated insoles. Stride parameters (stride length, stride rate) and shock acceleration parameters (head and tibial peak acceleration, shock magnitude, acceleration rate, and shock attenuation) were measured using two triaxial accelerometers in 38 runners at 3.33 m/s before and after a 15-min intense run while using the sock liner of the shoe (control condition), prefabricated insoles and custom-made insoles. No differences in shock accelerations were found between the custom-made and the control insoles. The prefabricated insoles increased the head acceleration rate (post-fatigue, p = 0.029) compared to the control condition. The custom-made reduced tibial (pre-fatigue, p = 0.041) and head acceleration rates (pre-fatigue and post-fatigue, p = 0.01 and p = 0.046) compared to the prefabricated insoles. Neither the stride nor the acceleration parameters were modified as a result of the intense run. In the present study, the acute use of insoles (custom-made, prefabricated) did not reduce shock accelerations compared to the control insoles. Therefore, their effectiveness at protecting against injuries associated with elevated accelerations is not supported and remains unclear.

## Introduction

Running is a type of physical activity that involves the athlete striking the ground about 600 times per kilometer [1,2]. Each foot strike during running there is a rapid deceleration of the lower-limb that results in a shock wave that is transmitted from the foot to the head [3]. On its way upwards to the head, this shock is partly absorbed by the ground, the running shoes and the musculoskeletal system in a process known as shock attenuation [4,5]. However, even though the musculoskeletal system is prepared to deal with each one of these contacts, their repetitive and cumulative effect on the human body could overload and fatigue the musculoskeletal system, especially of the lower leg, and lead to increased risk of overuse injuries such as patellofemoral pain syndrome, tibial stress fractures, plantar fasciitis, metatarsalgia and Achilles tendinitis [6–9]. In this sense, the analysis of the shock attenuation, the loading rate and the magnitude of the shock wave (also called impact or shock acceleration) during running is drawing the attention of the research community as a consequence of their relationship with tibial stress fractures [10,11], performance [4,5], and lower-limb comfort [12].

Repeated exposure to shock accelerations, as experienced by long distance runners, is believed to increase the incidence of injury as a result of the reduced ability of the musculoskeletal system to absorb these shock waves [13]. The ability of the musculoskeletal system to attenuate these accelerations decreases with fatigue, and therefore the articular cartilage and ligaments become more vulnerable to excessive loading stress loading [14]. In this sense, previous studies have observed that shock acceleration increases with speed and fatigue [2,15,16] and suggest that muscle fatigue also plays a role in overloading the musculoskeletal system leading to overuse injury [10].

Considering that shock accelerations are inherent to running, different strategies including modifying foot strike pattern [17], footwear [18,19], compressive garments [2], gait retraining [20] or insoles [21] are being investigated aiming to reduce such accelerations and therefore decrease the risk and frequency of injury in runners. Insoles are in-shoe devices widely prescribed by podiatrists to reduce or eliminate pathological stresses to the foot or other portions of the kinetic chain [22]. However, there is a great controversy between the effectiveness of over the shelf (prefabricated) insoles chosen by taking solely into account the individual’s foot size and custom-made insoles. On the one hand, custom-made insoles are devices built by a podiatrist from a three-dimensional representation of the individual’s foot and their use has been associated with pain relief [23,24], improved comfort [12], reduced plantar pressure [25], impact magnitude, and loading rate [26–28]. On the other hand, prefabricated insoles are mass-produced devices at a fraction of the cost of custom-made insoles and therefore it is not surprising that their use is expanding. Prefabricated cushioning insoles have also been associated with reduced plantar pressure [29], shock accelerations [27], impact forces, and loading rates [30]. However, there is a paucity of studies analysing the efficiency at attenuating the running-related accelerations of these types of insoles (custom-made vs prefabricated insoles) and compared to a control situation (sock liner of the running shoe) in the same population. Although individualised prescription is recognised to be a gold standard and it would be reasonable to expect that a custom-made insole adapted to the individual’s foot would better fulfil the runner’s expectations and provide more protection than a prefabricated insole [31], scientific evidence to demonstrate its benefits is needed. As a result, the aim of the present study was therefore to determine the effects of different insoles (custom-made, prefabricated, control) on stride and shock acceleration parameters before and after an intense run. It was hypothesised that the use of custom-made insoles would reduce shock accelerations compared to the control and prefabricated insoles. It was also hypothesised that runners would exhibit greater shock acceleration after the intense run independent of the insole condition.

## Methods

### Participants

A sample size of 34 participants was estimated using G*Power 3 software for a desired power of 80% from the results of published work which studied similar dependent variables [3,27]. As a result, thirty-eight recreational runners recruited from local running clubs participated in the study: 20 males and 18 females (29.8 ± 5.3 years; 170.3 ± 11.4 cm; 65.4 ± 10.1 kg, weekly running distance: 36.5 ± 7.2 km/week, best time in 10k race: 53.6 ± 9.4 min). Inclusion criteria were: I) no injuries in the last year, II) no previous lower-limb surgery in the last 3 years, III) no previous use of insoles, and IV) a training routine of at least 20 km / week. All runners provided written informed consent before participation. The study procedures complied with the Declaration of Helsinki and were approved by Fernando Alejo Verdú Pascual, acting secretary of the University ethics committee (Comité Ético de Investigación en Humanos de la Comisión de Ética en Investigación Experimental de la Universidad de Valencia, approval number H1411628681304).

### Insole conditions and customisation

**Fig 1:**
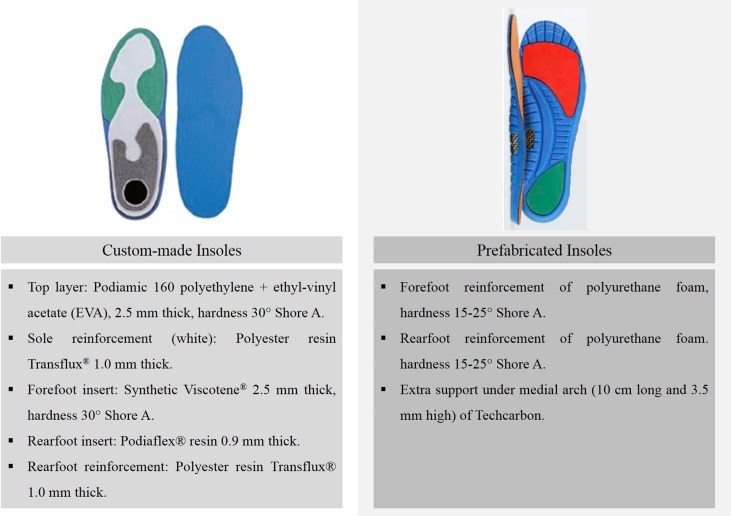
Characteristics of the insoles.

Participants carried out the study under three different conditions: the original sock liner of their running shoes (control), the prefabricated insoles (http://www.herbitas.com/plantilla-tecnoped-especial-running-p-4-50-2549/) (Tecnoped Run, Herbitas, Valencia, Spain) and the custom-made insoles (http://sidas.eurowintuecommerce.com/articulo/SPCTR-L-opctrunnings4243.html) (OPCT Run, Sidas S.L., Barcelona, Spain) (Fig 1). The custom-made insoles were initially the same in terms of material and properties, and their shape was afterwards customized based on the feet of the participants. For the customisation of the custom-made insoles, as described in detail previously [32], participants stood on a Printlab2 platform (Podiatech, Sidas Technologies, Voiron, France), which consisted of a pair of silicon vacuum bags that allowed an experienced podiatrist to create a plaster mould based on the plantar print of the participants taking into account their foot morphology. Afterwards, through a thermo-welding process, the custom-made insoles were warmed-up and adapted with a vacuum system (Mobilab2, Podiatech, Sidas Technologies, Voiron, France) to the exact shape of the heel, midfoot and forefoot of each participant using their individual feet plaster moulds. As a result, the insoles fitted perfectly to the plantar surface of the feet of the participants: feet with slightly lower medial arch resulted in insoles with lower height support in this area, whereas participants with slightly higher medial arch resulted in insoles with a higher support.

### Protocol

**Fig 2:**
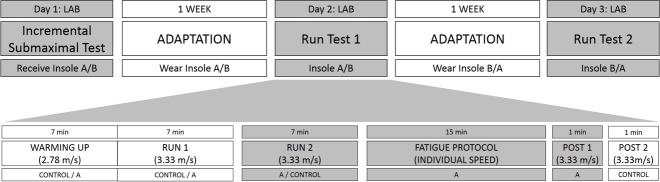
Study Design and Protocol.

Participants performed three running tests on different days and the total duration of the study was 2 weeks (Fig 2). All running tests were carried out on a treadmill (Excite Run 700, TechnoGymSpa, Gambettola, Italy). As described elsewhere [32], in the first laboratory session, participants performed an incremental test to determine their lactate threshold speed right below 4-mM blood lactate concentration [33]. This test involved a 5-min warm-up at 2.78 m/s followed by 0.56 m/s speed increments every 3 min. Blood samples were taken from the ear lobe at the end of each stage [33] and blood lactate concentration was determined using a Lactate Pro Analyzer (Arkay Factory Inc., Shiga, Japan). Blood lactate concentration was used as the physiological parameter for determining their individual lactate threshold speed as it is considered a useful tool to effectively predict exercise performance [34]. Then, the speed of the last stage before reaching 4 mM of blood lactate concentration was written down and, later on, in the laboratory sessions 2 and 3, was used as the fatiguing speed for the 15-min intense run. Following the incremental test of the first laboratory session, a pair of insoles (custom-made, prefabricated) was randomly given to each participant using a research randomizer program [35]. During the adaptation week, participants were asked to run 3 times using the assigned insoles and to lead their daily routine during this week (using the insoles with their sport footwear when going for a walk, in their leisure time, etc.) for adaptation purposes and return to the lab for session 2 (Fig 2: Run Test 1) after this adaptation week.

Since the use of insoles was a new situation for the participants, they were asked to wear their own running footwear during the adaptation week and throughout the tests in order to introduce no further changes in their running customary condition, as recommended by previous studies [36,37]. After the first familiarisation week, participants came to the lab to perform the laboratory session 2 (laboratory sessions 2 and 3 were identical with the only exception of the insole being used and measured, custom-made or prefabricated). In these laboratory sessions 2 and 3, participants performed a 7-min warm-up at 2.78 m/s with the sock liners of the shoe (control) or the study insoles of that session (custom-made or prefabricated) at random. Following the warm-up, participants ran for 7 min at 3.33 m/s and shock accelerations and stride parameters were measured within the last minute of the run. After this running bout, the insoles inside the footwear were replaced by the second condition (control or study insoles, depending on the initial order) and the 7-min run at 3.33 m/s was repeated in order to measure shock acceleration and stride parameters again. Afterwards, participants ran for 15 min (intense run) at their individual lactate threshold speed (4.04 ± 0.36 m/s). All participants were able to finish the intense run and the rating of perceived exertion between 6 and 20 [38] was also reported during the last minute of the run. Immediately after the intense run, acceleration and stride parameters were measured again during two 1-min runs at 3.33 m/s (post-fatigue control and post-fatigue insole conditions). The time between measurements was not longer than 1 minute (time needed to change the insoles inside the running shoes).

At the end of the laboratory session 2, participants received the second pair of study insoles (custom-made or prefabricated, depending on the initial randomisation) and repeated this running protocol with the control and the second pair of study insoles (laboratory session 3) after another adaptation week.

### Data collection

**Fig 3:**
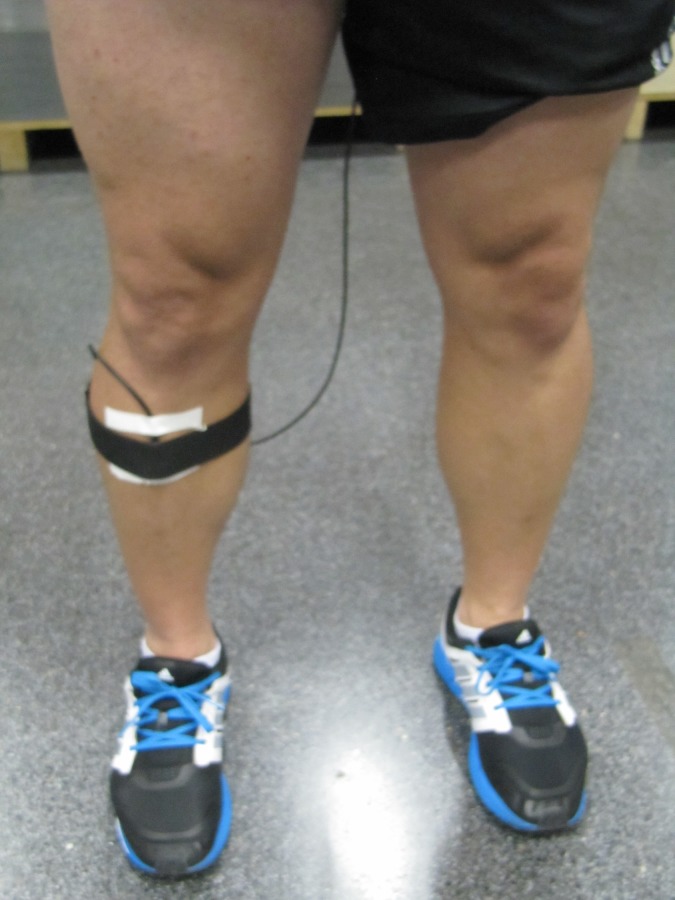
Accelerometer placement on the tibia.

Accelerations were measured during 10 seconds using two lightweight tri-axial accelerometers (Sportmetrics, Spain; mass: 2.5 g; dimensions: 40 mm × 22 mm × 12 mm; sampling frequency 500 Hz). As explained in detail elsewhere [2], the accelerometers were attached to the skin as tight as possible to the participants’ tolerance with double-sided adhesive tape and secured via elastic belts around the proximal anteromedial aspect of the tibia and around the forehead. The vertical axis of the accelerometer was aligned to be parallel to the long axis of the shank (Fig 3). Acceleration data were filtered (8-order low-pass digital Chebyshev type II filter, stop-band edge frequency 120 Hz, stop-band ripple 40 dB) [39] and analysed using Matlab (The Math Works Inc., Natick, MA, USA). From the acceleration signal, stride frequency was calculated as the time between consecutive leg impacts, whereas stride length was obtain by dividing running speed by stride rate [5]. On the other hand, the following acceleration parameters were also calculated [2]: head and tibia peak acceleration (maximal amplitude), acceleration magnitude (difference between the positive and the negative peak), acceleration rate (slope from ground contact to peak acceleration), and shock attenuation (reduction in peak acceleration from the tibia to the head as a percentage of the head acceleration).

### Statistical analysis

A commercial statistical package (SPSS 18.0, SPSS Inc., Chicago, IL, USA) was used for statistical analyses. After checking the normality of the variables (Kolmogorov–Smirnov), a descriptive analysis of the data was performed. The sphericity assumption was verified by the Mauchly test. Then, a two-way repeated-measures ANOVA with insole (control, prefabricated, custom-made) and fatigue (pre- and post- intense run) as intra-subject factors and acceleration and stride parameters as dependent variables was performed. Bonferroni post-hoc was carried out to provide details as to the whereabouts of significant differences. Significance was set at α = 0.05. Data are presented as mean ± 95% confidence intervals (95% CI).

## Results

### Effect of the insole condition

The different insoles did not influence stride rate and stride length (*p* > 0.05) (Table 1). However, the insole conditions did affect the shock accelerations during running (Table 2). In the pre-fatigue state, the use of custom-made insoles reduced the head acceleration rate (*p* = 0.041, mean difference: 6.3, 95%CI mean difference: 0.21–12.48) and the tibial acceleration rate (*p* = 0.014, mean difference: 85.38, 95%CI mean difference: 14.56–156.20) compared to the prefabricated insoles. Moreover, in the post-fatigue state, the prefabricated insoles increased the head acceleration rate compared to the custom-made (*p* = 0.046, mean difference: 6.84, 95%CI mean difference: 0.11–13.59) and the control insoles (*p* = 0.029, mean difference: 6.97, 95%CI mean difference: 0.56–13.38). No difference was observed between the custom-made and the control insoles for any of the parameters analysed (*p* > 0.05).

**Table 1:** Mean (95% confidence intervals) of the stride parameters for the different insole conditions and fatigue state.

	PRE			POST		
	Control	Prefabricated	Custom-made	Control	Prefabricated	Custom-made
Stride Rate (stride/s)	1.41 (1.39–1.44)	1.42 (1.39–1.44)	1.41 (1.37–1.44)	1.42 (1.39–1.44)	1.42 (1.39–1.44)	1.37 (1.28–1.49)
Stride Length (m/stride)	2.36 (2.32–2.41)	2.36 (2.31–2.40)	2.36 (2.31–2.41)	2.36 (2.31–2.41)	2.36 (2.31–2.41)	2.37 (2.32–2.42)

PRE: pre-fatigue; POST: post-fatigue. No significant difference was found between the pre-fatigue and the post-fatigue values.

**Table 2:** Mean (95% confidence intervals) of the acceleration parameters for the different insole conditions and fatigue state.

	PRE			POST		
	Control	Prefabricated	Custom-made	Control	Prefabricated	Custom-made
Max Tibia (G)	7.89 (7.00–8.78)	8.13 (7.15–9.11)	7.69 (6.93–8.44)	7.75 (6.73–8.77)	8.59 (7.55–9.63)	7.96 (6.91–9.00)
Max Head (G)	2.37 (2.20–2.54)	2.38 (2.15–2.60)	2.31 (2.13–2.49)	2.25 (2.01–2.48)	2.34 (2.06–2.63)	2.27 (2.08–2.47)
Magnitude Tibia (G)	8.54 (7.63–9.46)	8.63 (7.56–9.69)	8.61 (7.79–9.44)	8.50 (7.48–9.52)	9.31 (8.19–10.42)	9.05 (7.96–10.13)
Magnitude Head (G)	2.43 (2.26–2.60)	2.41 (2.19–2.63)	2.41 (2.23–2.60)	2.31 (2.09–2.53)	2.38 (2.11–2.65)	2.36 (2.17–2.56)
Tibia Rate (G/s)	272.28 (200.67–343.90)	319.99 (236.96–403.02)	234.61*^b^ (173.53–295.70)	257.03 (186.06–328.01)	340.06 (237.50–442.61)	287.50 (187.58–387.41)
Head Rate (G/s)	55.05 (48.96–61.14)	58.33 (50.40–66.26)	51.98*^b^ (44.93–59.04)	51.34 (43.86–58.82)	58.31*^a^ (48.73–67.90)	51.47*^b^ (44.06–58.87)
Attenuation (%)	66.43 (62.52–70.34)	67.37 (62.94–71.80)	65.78 (60.33–71.23)	66.82 (61.71–71.92)	70.55 (66.89–74.20)	64.85 (55.54–74.16)

PRE: pre-fatigue; POST: post-fatigue.

*^a^ *P* < .05. significant difference compared to control insoles for the matching fatigue condition.

*^b^ *P* < .05. significant difference compared to prefabricated insoles for the matching fatigue condition. No significant difference was found between the pre-fatigue and the post-fatigue values.

### Effect of the intense run

Participants considered that the intense protocol was ‘Hard’ as they reported a rating of perceived exertion of 14.34 (13.40–15.42) within the last minute of the intense run. Stride rate and stride length were not influenced by the intense run (p > 0.05) (Table 1). Similarly, the intense run did not modify any of the shock acceleration parameters measured in the study (*p* > 0.05) (Table 2).

The effect of the insole intervention on the stride and shock accelerations parameters was not modified by the fatigue state, as no significant interaction (*p* > 0.05) was observed between the two factors (insole, fatigue).

## Discussion

This study analysed the effects of prefabricated and custom-made insoles on stride and shock acceleration parameters before and after an intense run. Our main finding suggests that even though the use of custom-made insoles reduced the acceleration rate at the tibia and head compared to prefabricated insoles, no major differences were observed between the study insoles (custom-made, prefabricated) and the control insoles.

Prolonged and elevated magnitudes of shock accelerations have been associated with increased risk of injuries [11]. The use of different strategies including gait retraining, compressive garments or cushioned shoes or insoles have aimed to reduce these shock accelerations during running [2,20,27,30,40]. In the present study, it was hypothesised that custom-made insoles would reduce the shock acceleration experienced by the runner compared to the prefabricated and the control insoles. However, this hypothesis was only partly supported as the use of custom-made insoles only led to a lower acceleration rate compared to prefabricated insoles, whereas no differences with the control condition were observed. Moreover, no alterations of the tibial and head peak accelerations were observed when running with the study insoles (custom-made, prefabricated) compared to the control condition, and therefore these findings question the efficacy of insoles when aiming to reduce shock accelerations during running.

The use of insoles has been suggested as a strategy to reduce the shock accelerations associated with running, thereby decreasing the risk of overuse injuries [27,41]. However, while most of the previous studies analysed the effect of insoles compared to a control situation (running with insoles versus running without insoles) [27,30,42], to the authors’ knowledge this is the first study to analyse the effect of custom-made insoles before and after an intense run on impact accelerations during running compared to prefabricated and control insoles.

In the present study neither the acceleration peaks nor the acceleration magnitudes on the tibia and head were altered when running with insoles compared to the control condition. Although this result is in accordance with Laughton et al. [42], who did not find differences in tibial peak accelerations when running with and without customised insoles; it is also in contrast with two previous studies, who observed reduced tibial peak accelerations when running with cushioned insoles [21] and semi-rigid prefabricated insoles inside military boots [30]. One of the reasons that may explain the differences among studies is the cushioning system of the footwear. In this sense, running shoes have inherently greater shock attenuation properties than street shoes or military boots, and consequently the overall effect of the shoe-insole complex may vary depending on the footwear [41]. Another reason that will likely explain the controversy among studies is the different materials and the thickness of the layers used to build the insoles of each study. In this sense, in contrast with the polyethylene + EVA (custom-made) and polyurethane foam with Techcarbon (prefabricated) of the insoles used in this study, previous studies have used insoles based on a number of materials such as polyurethane foam + Poron foam [21], Trocellen foam with polypropylene [30], or suborthelene covered with a neoprene pad [42]. As a result, the behaviour of the different materials against vibrations and accelerations may explain the differences between studies.

Recent studies are emphasising the role of loading rate rather than peak acceleration values when analysing the effects of the resulting shock wave following exercise on the musculoskeletal system [43,44]. Repetitive, rapidly applied loads are more associated with joint degeneration than slowly applied loads of equal or even greater magnitudes [45] and a recent study has found a positive relationship between greater acceleration rate and stress fractures [11]. Moreover, loading rate may describe better than the acceleration magnitude the capacity of the cushion structure (footwear, insole) to reduce the rate at which the shock acceleration is transmitted to the lower extremity and may be a better indicator of cushioning performance [46]. In the present study, it was observed that the prefabricated insoles increased by 27% and 11% the tibial and head acceleration rates compared to the custom-made insoles and by 12% the head acceleration rates compared to the control condition, which contrasts with a recent study who found a reduction of the tibial acceleration rate during running with insoles compared to running without insoles [42]. The differences between studies may be explained by the deformation of the materials of the insoles, as it has been previously suggested that the materials of shoe-insole complex determine the spring stiffness of the footwear-insole-foot system and ultimately influence their behaviour against accelerations during running [47–50]. Therefore, and taking into account that the acceleration rates may represent the cushioning performance of the structure and influence the risk of overuse running injuries, the use of prefabricated insoles as a protective mechanism against accelerations during running is not supported. However, as custom-made insoles decreased both the tibial and the head acceleration rates compared to the prefabricated insoles, if a runner would need to use insoles for a given biomechanical reason (comfort, motion control, plantar redistribution), the use of custom-made insoles would behave better at attenuating shock accelerations than the prefabricated insoles and could be more effective as a protective strategy to reduce the risk of overuse running-related injuries or as a conservative treatment for the rehabilitation of runners after an overuse running injury, similar to those studies observing lower shock accelerations resulting from gait retraining [20,51]. However, this hypothesis remains just a speculation and future studies should investigate it.

On the other hand, no difference between the custom-made and the control insoles was observed for any of the shock acceleration parameters, which indicates that even though the use of custom-made insoles has been observed to effectively relieve pain [23,24], improve comfort [12], and redistribute plantar pressure [25], their role as a shock-absorbing strategy during running is not supported either. Only two studies have observed a reduction of shock acceleration during running with insoles compared to running without insoles [27,41]. However, the insoles used in those studies were described as cushioned insoles (3–6 mm thick with foam cover) [27] and shock-absorbing insoles (1–6 mm thick with foam support) [41]. Whereas the insoles in the present study were made of harder and stiffer materials and may have stabilise better the movement of the rearfoot. Taking into account that foot pronation is considered a shock-absorbing mechanism [52,53], it could be speculated that the control provided by the use of insoles could reduce the pronation of the foot, thereby reducing the efficiency of this shock-absorption mechanism and lead to greater shock accelerations. However, foot pronation was not measured in this study and this speculation needs to be further investigated.

Of special relevance is the recent publication by Nigg et al.[54], who stated that there is still no evidence to confirm the relationship between certain factors that traditionally were believed to increase injury risk such as pronation or shock accelerations and the probability of suffering a running-related injury. These authors indicate that studies on this field to support this association are insufficient and those who observed a relationship between shock accelerations and injury risk had a small sample size. Therefore, there is still controversy nowadays regarding the role that accelerations play during running and their effect on the human body overtime. As a result, future studies analysing the effects or long-term exposure to shock accelerations on the human body are encouraged to throw some light into this interesting matter.

The majority of the running-related studies are conducted in a non-exerted state. Although difficult, analysing the effects of the fatigue is important because it is a regular state experienced by all runners and it is when the athlete is fatigue that most overuse running-related injuries are thought to occur [2,55]. In the present study it was hypothesised that the fatigue state provoked by the intense run would increase shock acceleration. However, our results showed no changes in peak acceleration and acceleration rate with the development of the fatigue state. Previous studies have found an increase [2,15,16,56] as well as a reduction [57] of shock accelerations with fatigue. These authors suggested that a change in the attenuation properties of the body as a result of muscle fatigue could be due to the loss of the shock-absorbing capacity of muscles or to alterations in the lower extremity kinematics to compensate for the change in muscle ability [15,58]. In this sense, a decrease in stride rate leading to a greater shock acceleration was reported after a fatigue run [16,56]. These authors suggested that the alteration of the ‘optimal’ stride rate could have influenced shock transmission. However, the runners in our study, in agreement with Mercer et al.[58], did not make any adjustments to stride rate in response to fatigue. This result may indicate that runners in the present study were able to maintain their optimal stride rate and it could explain why the shock accelerations were not modified after the intense run. Discrepancies in the shock acceleration behaviour after the intense run can be attributed to the differences in the fatigue protocols used between studies. In the current study, in order to have a greater ecological validity, participants run for 36 minutes (21 minutes resulting from the pre-fatigue running conditions plus 15 minutes of the intense run) at a training pace, which is a fatigue state more commonly reached within the recreational running population, rather than an incremental running protocol to exhaustion. On the other hand, other studies measured shock acceleration on a runway after a 20-min and a 40-min run [33], on a treadmill after a 30-min run [16,56], or throughout an increasing protocol until exhaustion [15,58]. Thus, the actual level of fatigue attained by the participants and the type of exercise chosen to reach the fatigue state (short protocols at high intensity versus longer protocols at lower intensity) may account for the inconsistent results observed in the literature.

Running on a treadmill could be considered a limitation of the study. Even though a treadmill was used in order to better control some variables (running speed, hardness and slope of the running surface), running on a treadmill could lead to different running biomechanics compared to overground running [3]. Moreover, the running pattern of the athletes (rearfoot, midfoot, forefoot) and the cushioning system of the athlete’s footwear was not controlled (standard shoes were not provided) in order not to alter further their running customary conditions, but these factors may influence shock accelerations and future studies should look at these parameters while controlling running pattern and footwear. The two models of insoles (custom-made, prefabricated) were chosen based on their popularity among runners and podiatrists. While these result are interesting because they come from analysing two very popular types of insoles, caution is advised when interpreting these results as the differences in materials and stiffness of the insoles and running shoes were not taken into account, which may have influenced the results. As a result, future studies should control the materials and properties of the insoles and running shoes. Finally, participants in our study reported an average RPE value of 14 (Hard) after the intense run, which indicates that the intense run may have not been fatiguing enough to provoke some of the biomechanical adaptations observed in previous studies. Therefore, in future studies it would be of interest to investigate the effects of custom-made and prefabricated insoles on shock acceleration during overground running or after more extenuating running tests in order to provide a better insight into the shock attenuation mechanisms of these types of insoles and their potential role as an injury-prevention strategy.

## Conclusion

This study demonstrated that the acute use of insoles (both custom-made and prefabricated) did not reduce shock accelerations compared to the control condition. However, it was observed that custom-made insoles reduced tibial and head acceleration rate compared to prefabricated insoles. Although the effectiveness of insoles at reducing shock accelerations during running remains unclear, the custom-made insoles led to lower shock acceleration rates than the prefabricated insoles and therefore showed a better shock attenuation behaviour.

## Supporting information

S1 Table Raw data of the study. (XLSX)

## References

[1] Guo L-Y, Su F-C, Yang C-H, Wang S-H, Chang J-J, Wu W-L, et al Effects of speed and incline on lower extremity kinematics during treadmill jogging in healthy subjects. Biomed Eng Appl Basis Commun. 2006; 73–79.

[2] Lucas-Cuevas AG, Priego-Quesada JI, Aparicio I, Giménez JV, Llana-Belloch S, Pérez-Soriano P. Effect of 3 Weeks Use of Compression Garments on Stride and Impact Shock during a Fatiguing Run. Int J Sports Med. 2015;36: 826–831. 10.1055/s-0035-1548813 26090880

[3] García-Pérez JA, Pérez-Soriano P, Llana-Belloch S, Lucas-Cuevas AG, Sánchez-Zuriaga D. Effects of treadmill running and fatigue on impact acceleration in distance running. Sports Biomech. 2014;13: 259–266. 10.1080/14763141.2014.909527 25325770

[4] Derrick TR. The effects of knee contact angle on impact forces and accelerations. Med Sci Sports Exerc. 2004;36: 832–837. 15126718 10.1249/01.mss.0000126779.65353.cb

[5] Mercer JA, Vance J, Hreljac A, Hamill J. Relationship between shock attenuation and stride length during running at different velocities. Eur J Appl Physiol. 2002;87: 403–408. 10.1007/s00421-002-0646-9 12172880

[6] Tessutti V, Trombini-Souza F, Ribeiro AP, Nunes AL, Sacco I de CN. In-shoe plantar pressure distribution during running on natural grass and asphalt in recreational runners. J Sci Med Sport. 2010;13: 151–155. 10.1016/j.jsams.2008.07.008 18977172

[7] Foch E, Reinbolt JA, Zhang S, Fitzhugh EC, Milner CE. Associations between iliotibial band injury status and running biomechanics in women. Gait Posture. 2015;41: 706–710. 10.1016/j.gaitpost.2015.01.031 25701012

[8] Nielsen RO, Rønnow L, Rasmussen S, Lind M. A prospective study on time to recovery in 254 injured novice runners. PloS One. 2014;9: e99877 10.1371/journal.pone.0099877 24923269 PMC4055729

[9] Van Ginckel A, Thijs Y, Hesar NGZ, Mahieu N, De Clercq D, Roosen P, et al Intrinsic gait-related risk factors for Achilles tendinopathy in novice runners: a prospective study. Gait Posture. 2009;29: 387–391. 10.1016/j.gaitpost.2008.10.058 19042130

[10] Clinghan R, Arnold GP, Drew TS, Cochrane LA, Abboud RJ. Do you get value for money when you buy an expensive pair of running shoes? Br J Sports Med. 2008;42: 189–193. 10.1136/bjsm.2007.038844 17932096

[11] Milner CE, Ferber R, Pollard CD, Hamill J, Davis IS. Biomechanical factors associated with tibial stress fracture in female runners. Med Sci Sports Exerc. 2006;38: 323–328. 10.1249/01.mss.0000183477.75808.92 16531902

[12] Lucas-Cuevas AG, Pérez-Soriano P, Priego-Quesada JI, Llana-Belloch S. Influence of foot orthosis customisation on perceived comfort during running. Ergonomics. 2014;57: 1590–1596. 10.1080/00140139.2014.938129 25009959

[13] Mizrahi J, Daily D. Modeling the Foot-Strike Event in Running Fatigue via Mechanical Impedances In: Goswami T, editor. Injury and Skeletal Biomechanics. InTech; 2012.

[14] Whittle MW. Generation and attenuation of transient impulsive forces beneath the foot: a review. Gait Posture. 1999;10: 264–275. 10567759 10.1016/s0966-6362(99)00041-7

[15] Derrick TR, Dereu D, McLean SP. Impacts and kinematic adjustments during an exhaustive run. Med Sci Sports Exerc. 2002;34: 998–1002. 12048328 10.1097/00005768-200206000-00015

[16] Mizrahi J, Verbitsky O, Isakov E. Fatigue-related loading imbalance on the shank in running: a possible factor in stress fractures. Ann Biomed Eng. 2000;28: 463–469. 10870903 10.1114/1.284

[17] Giandolini M, Horvais N, Rossi J, Millet GY, Samozino P, Morin J-B. Foot strike pattern differently affects the axial and transverse components of shock acceleration and attenuation in downhill trail running. J Biomech. 2016;49: 1765–1771. 10.1016/j.jbiomech.2016.04.001 27087676

[18] Chambon N, Delattre N, Guéguen N, Berton E, Rao G. Is midsole thickness a key parameter for the running pattern? Gait Posture. 2014;40: 58–63. 10.1016/j.gaitpost.2014.02.005 24636223

[19] TenBroek T, Rodrigues R, Frederick E, Hamill J. Effects of unknown footwear midsole thickness on running kinematics within the initial six minutes of running. Footwear Sci. 2013;5: 27–37.

[20] Crowell HP, Davis IS. Gait retraining to reduce lower extremity loading in runners. Clin Biomech. 2011;26: 78–83. 10.1016/j.clinbiomech.2010.09.003 PMC3014399 20888675

[21] O’Leary K, Vorpahl KA, Heiderscheit B. Effect of cushioned insoles on impact forces during running. J Am Podiatr Med Assoc. 2008;98: 36–41. 18202332 10.7547/0980036

[22] Hunter S, Dolan MG, Davis JM. Foot orthotics in therapy and sport. Champaign, IL: Human Kinetics; 1995.

[23] Hirschmüller A, Baur H, Müller S, Helwig P, Dickhuth H-H, Mayer F. Clinical effectiveness of customised sport shoe orthoses for overuse injuries in runners: a randomised controlled study. Br J Sports Med. 2011;45: 959–965. 10.1136/bjsm.2008.055830 19679575

[24] Gijon-Nogueron G, Cortes-Jeronimo E, Cervera-Marin JA, Diaz-Mohedo E, Lopezosa-Reca E, Fernandez-Sanchez M, et al The effects of custom-made foot orthosis using the Central Stabilizer Element on foot pain. Prosthet Orthot Int. 2014. 10.1177/0309364614531012 24812119

[25] Lee Y-C, Lin G, Wang M-JJ. Evaluating insole design with joint motion, plantar pressure and rating of perceived exertion measures. Work J. 2012;41: 1114–1117. 10.3233/WOR-2012-0290-1114 22316868

[26] Dixon SJ, Waterworth C, Smith CV, House CM. Biomechanical analysis of running in military boots with new and degraded insoles. Med Sci Sports Exerc. 2003;35: 472–479. 10.1249/01.MSS.0000053733.64049.27 12618578

[27] O’Leary K, Vorpahl KA, Heiderscheit B. Effect of cushioned insoles on impact forces during running. J Am Podiatr Med Assoc. 2008;98: 36–41. 18202332 10.7547/0980036

[28] Creaby MW, May K, Bennell KL. Insole effects on impact loading during walking. Ergonomics. 2011;54: 665–671. 10.1080/00140139.2011.592600 21770753

[29] Hinz P, Henningsen A, Matthes G, Jäger B, Ekkernkamp A, Rosenbaum D. Analysis of pressure distribution below the metatarsals with different insoles in combat boots of the German Army for prevention of march fractures. Gait Posture. 2008;27: 535–538. 10.1016/j.gaitpost.2007.06.005 17692523

[30] Dixon SJ. Influence of a commercially available orthotic device on rearfoot eversion and vertical ground reaction force when running in military footwear. Mil Med. 2007;172: 446–450. 17484322 10.7205/milmed.172.4.446

[31] Gijon-Nogueron G, Cortes-Jeronimo E, Cervera-Marin JA, García-de-la-Peña R, Benhamu-Benhamu S, Luque-Suarez A. Foot orthoses custom-made by vacuum forming on the non-load-bearing foot: preliminary results in male children with calcaneal apophysitis (Sever’s disease). Prosthet Orthot Int. 2013;37: 495–498. 10.1177/0309364613482844 23585194

[32] Lucas-Cuevas AG, Pérez-Soriano P, Llana-Belloch S, Macián-Romero C, Sánchez-Zuriaga D. Effect of custom-made and prefabricated insoles on plantar loading parameters during running with and without fatigue. J Sports Sci. 2014;32: 1712–1721. 10.1080/02640414.2014.915422 24823258

[33] Clansey AC, Hanlon M, Wallace ES, Lake MJ. Effects of fatigue on running mechanics associated with tibial stress fracture risk. Med Sci Sports Exerc. 2012;44: 1917–1923. 10.1249/MSS.0b013e318259480d 22525776

[34] McArdle WD, Katch FI, Katch VL. Essentials of Exercise Physiology. Lippincott Williams & Wilkins; 2006.

[35] Urbaniak GC, Plous S. Research Randomizer [Computer software]. Retrieved from http://www.randomizer.org/.

[36] Nagel A, Fernholz F, Kibele C, Rosenbaum D. Long distance running increases plantar pressures beneath the metatarsal heads: a barefoot walking investigation of 200 marathon runners. Gait Posture. 2008;27: 152–155. 10.1016/j.gaitpost.2006.12.012 17276688

[37] Weist R, Eils E, Rosenbaum D. The influence of muscle fatigue on electromyogram and plantar pressure patterns as an explanation for the incidence of metatarsal stress fractures. Am J Sports Med. 2004;32: 1893–1898. 15572318 10.1177/0363546504265191

[38] Borg GA. Psychophysical bases of perceived exertion. Med Sci Sports Exerc. 1982;14: 377–381. 7154893

[39] Parks TW, Burrus CS. Digital Filter Design. New York, NY, USA: Wiley-Interscience; 1987.

[40] Butler RJ, Hamill J, Davis I. Effect of footwear on high and low arched runners’ mechanics during a prolonged run. Gait Posture. 2007;26: 219–225. 10.1016/j.gaitpost.2006.09.015 17055729

[41] Windle CM, Gregory SM, Dixon SJ. The shock attenuation characteristics of four different insoles when worn in a military boot during running and marching. Gait Posture. 1999;9: 31–37. 10575068 10.1016/s0966-6362(99)00002-8

[42] Laughton C, Davis I, Hamill J. Effect of Strike Pattern and Orthotic Intervention on Tibial Shock During Running. J Appl Biomech. 2003;19: 153–168.

[43] Dixon SJ, Collop AC, Batt ME. Surface effects on ground reaction forces and lower extremity kinematics in running. Med Sci Sports Exerc. 2000;32: 1919–1926. 11079523 10.1097/00005768-200011000-00016

[44] Zadpoor AA, Nikooyan AA. The relationship between lower-extremity stress fractures and the ground reaction force: a systematic review. Clin Biomech Bristol Avon. 2011;26: 23–28. 10.1016/j.clinbiomech.2010.08.005 20846765

[45] Radin EL, Yang KH, Riegger C, Kish VL, O’Connor JJ. Relationship between lower limb dynamics and knee joint pain. J Orthop Res Off Publ Orthop Res Soc. 1991;9: 398–405. 10.1002/jor.1100090312 2010844

[46] Aguinaldo A, Mahar A. Impact loading in running shoes with cushioning column systems. J Appl Biomech. 2003;19: 353–360.

[47] Cavanagh PR. The running shoe book. Mountain View, CA: Anderson World; 1980.

[48] Nigg BM, Bahlsen HA, Luethi SM, Stokes S. The influence of running velocity and midsole hardness on external impact forces in heel-toe running. J Biomech. 1987;20: 951–959. 3693376 10.1016/0021-9290(87)90324-1

[49] Campbell G, Newell E, McLure M. Compression testing of foamed plastics and rubbers for use as orthotic show insoles. Prosthet Orthot Int. 1982;6: 48–52. 10.3109/03093648209167741 7079112

[50] Campbell GJ, McLure M, Newell EN. Compressive behavior after simulated service conditions of some foamed materials intended as orthotic shoe insoles. J Rehabil Res Dev. 1984;21: 57–65. 6530678

[51] Crowell HP, Milner CE, Hamill J, Davis IS. Reducing impact loading during running with the use of real-time visual feedback. J Orthop Sports Phys Ther. 2010;40: 206–213. 10.2519/jospt.2010.3166 20357417

[52] Grech C, Formosa C, Gatt A. Shock attenuation properties at heel strike: Implications for the clinical management of the cavus foot. J Orthop. 2016;13: 148–151. 10.1016/j.jor.2016.03.011 27408486 PMC4919312

[53] Pratt DJ. Mechanisms of shock attenuation via the lower extremity during running. Clin Biomech. 1989;4: 51–57. 10.1016/0268-0033(89)90068-5 23915960

[54] Nigg BM, Baltich J, Hoerzer S, Enders H. Running shoes and running injuries: mythbusting and a proposal for two new paradigms: “preferred movement path” and “comfort filter.” Br J Sports Med. 2015; bjsports-2015-095054. 10.1136/bjsports-2015-095054 26221015

[55] Hreljac A. Impact and overuse injuries in runners. Med Sci Sports Exerc. 2004;36: 845–849. 15126720 10.1249/01.mss.0000126803.66636.dd

[56] Verbitsky O, Mizrahi J, Voloshin A, Treiger J, Isakov E. Shock Transmission and Fatigue in Human Running. J Appl Biomech. 1998;14: 300–311. 10.1123/jab.14.3.300 28121250

[57] Gerlach KE, White SC, Burton HW, Dorn JM, Leddy JJ, Horvath PJ. Kinetic changes with fatigue and relationship to injury in female runners. Med Sci Sports Exerc. 2005;37: 657–663. 15809566 10.1249/01.mss.0000158994.29358.71

[58] Mercer JA, Bates BT, Dufek JS, Hreljac A. Characteristics of shock attenuation during fatigued running. J Sports Sci. 2003;21: 911–919. 10.1080/0264041031000140383 14626370

